# Clinical and molecular characterization of 10 Chinese children with congenital adrenal hyperplasia due to 11beta-hydroxylase deficiency

**DOI:** 10.1007/s12519-023-00739-1

**Published:** 2023-07-24

**Authors:** Wen-Li Lu, Xiao-Yu Ma, Jiao Zhang, Jun-Qi Wang, Ting-Ting Zhang, Lei Ye, Yuan Xiao, Zhi-Ya Dong, Wei Wang, Shou-Yue Sun, Chuan-Yin Li, Rong-Gui Hu, Guang Ning, Li-Dan Zhang

**Affiliations:** 1 Department of Pediatrics, Ruijin Hospital, Shanghai Jiao Tong University School of Medicine, No. 197, Ruijin 2nd Rd, Huangpu District, Shanghai, 200025 China; 2 Department of Pediatric Genetic and Metabolic Endocrinology, West China Second University Hospital,Sichuan University, No. 20, Section 3, Renmin South Road, Sichuan, 610041 China; 3 Department of Endocrine and Metabolic Diseases, Shanghai Clinical Center for Endocrine and Metabolic Diseases, Ruijin Hospital, Shanghai Jiao Tong University School of Medicine, Shanghai, 200025 China; 4 Cancer Center, School of Medicine, Shanghai Tenth People’s Hospital, Tongji University, Yanchang Zhong Lu 301St Rd, Jing’an District, Shanghai, 200031 China

**Keywords:** 11β-hydroxylase deficiency, CYP11B1, Enzymatic activity, Phenotype-genotype correlation, Three-dimensional protein simulations

## Abstract

**Background:**

The clinical manifestations of nonclassical 11beta-hydroxylase deficiency are very similar to those of non-classical 21-hydroxylase deficiency. For this study, we investigated the relationship between the clinical and molecular features of congenital adrenal hyperplasia caused by 11beta-hydroxylase deficiency and reviewed the related literature, which are expected to provide assistance for the clinical diagnosis and analysis of congenital adrenal hyperplasia.

**Methods:**

Clinical data for 10 Chinese patients diagnosed with congenital adrenal hyperplasia in our hospital from 2018 to 2022 were retrospectively analyzed. We examined the effects of gene mutations on protease activity and constructed three-dimensional structure prediction models of proteins.

**Results:**

We describe 10 patients with 11beta-hydroxylase gene mutations (*n* = 5, 46,XY; *n* = 5, 46,XX), with 10 novel mutations were reported. Female patients received treatment at an early stage, with an average age of 2.08 ± 1.66 years, whereas male patients received treatment significantly later, at an average age of 9.77 ± 3.62 years. The most common CYP11B1 pathogenic variant in the Chinese population was found to be c.1360C > T. All mutations lead to spatial conformational changes that affect protein stability.

**Conclusions:**

Our study found that there was no significant correlation between each specific mutation and the severity of clinical manifestations. Different patients with the same gene pathogenic variant may have mild or severe clinical manifestations. The correlation between genotype and phenotype needs further study. Three-dimensional protein simulations may provide additional support for the physiopathological mechanism of genetic mutations.

## Introduction

Congenital adrenal hyperplasia (CAH) is a group of autosomal recessive diseases. The most common form of CAH originates from steroid 21-hydroxylase deficiency (21OHD), which accounts for about 90%–95% of all cases, followed by 11beta-hydroxylase deficiency (11βOHD) (OMIM 202010), which accounts for about 5%–8% of cases [1]. The incidence of 11β-OHD in the total population is approximately one in 100,000–200,000 [2, 3], and it is common in the Israeli population of Jewish origin in the Middle East and North Africa, affecting 1:5000–7000 live births [4]. 11β-OHD is caused by mutations in the cytochrome P450 family 11 subfamily B member 1 (*CYP11B1*) gene. *CYP11B1* mutation leads to a reduction in the conversion of 11 deoxycorticosterone (DOC) to corticosterone and 11 deoxycorticosterol to cortisol, which leads to an increase in the secretion of adrenocorticotropic hormone (ACTH) and the accumulation of steroid precursors. Thus, 11β-OHD is characterized by increased serum concentrations of 11β-deoxycorticosterone, 17-hydroxyprogesterone (17-OHP) and androgens, resulting in hypertension with low renin levels, hypokalemia and genital ambiguity in affected individuals with a 46,XX karyotype. The milder nonclassical form is very similar to the nonclassical form of 21-OHD and is characterized by mild virilization, irregular menstruation, and male precocious puberty; hypertension does not occur frequently. According to the Human Gene Mutation Database (HGMD, www.hgmd.cf.ac.uk), a total of 155 *CYP11B1* gene mutations were included, of which missense and nonsense mutations are among the most common types, and their effects on enzyme activity have been studied mainly through three-dimensional protein structure simulation and in vitro protein expression experiments.

Here, we report the clinical and molecular characterization of 10 Chinese patients with CAH due to 11β-OHD. Novel mutations and three previously reported but not functionally studied mutations were explored in vitro to monitor the residual enzyme activity and to analyze the correlation between the genotype and phenotype of the new mutations, providing help for clinical diagnosis and analysis of 11β-OHD. Three-dimensional protein simulations may provide additional support for the physiopathological mechanism of genetic mutations.

## Methods

### Subjects

Ten patients from unrelated Chinese families who had no history of consanguinity were enrolled. Some patients were first diagnosed with congenital adrenocortical insufficiency or CAH in other hospitals and then transferred to Ruijin Hospital for detailed examination. We gathered the documented clinical characteristics and blood biochemical examinations during each patient’s initial admission to the hospital (Ruijin Hospital or others) for CAH. The study was conducted in accordance with the Declaration of Helsinki (as revised in 2013). The study was approved by the Ruijin Hospital Ethics Committee, Shanghai Jiao Tong University School of Medicine, and all the children and their guardians signed informed consent.

### DNA sequence analysis of *CYP11B1*

Genomic DNA was extracted from peripheral blood using a DNA extraction kit (Qiagen, Hilden Germany) according to the manufacturer’s instructions. *CYP11B1* gene (NM_000497.3) primers designed for polymerase chain reaction (PCR) were obtained from GenBank (Table 1). PCR products from primer pairs were resolved by 2% agarose gel electrophoresis and sequenced using an automated sequencer (ABI PRISM 3100 Genetic Analyzer; Applied Biosystems by Life Technologies, Carlsbad, CA, USA). Positive sequences were compared to the NCBI entry for *CYP11B1* to confirm the success of site-directed mutagenesis.

**Table 1:** Design of *CYP11B1* gene primers

Numbers	Primers	Sequences
1	400G > C-F	GCGTGTTCTTGCTGAATcGGCCTGAATGGCGC
	400G > C-R	GCGCCATTCAGGCCgATTCAGCAAGAACACGC
2	427C > T-F	GCTTCAACCGATTGtGGCTGAATCCAG
	427C > T-R	CTGGATTCAGCCaCAATCGGTTGAAGC
3	456C > G-F	GCTGTCGCCCAAgGCTGTGCAGAGG
	456C > G-R	CCTCTGCACAGCcTTGGGCGACAGC
4	799G > T-F	GCATCTTCCAGTACtGCGACAACTGTATCC
	799G > T-R	GGATACAGTTGTCGCaGTACTGGAAGATGC
5	896 T > C-F	CTGTTGAATGCGGAACcGTCGCCAGATGC
	896 T > C-R	GCATCTGGCGACgGTTCCGCATTCAACAG
6	945C > A-F	CTCACTGCAGGGAGaGTGGACACGACG
	945C > A-R	CGTCGTGTCCACtCTCCCTGCAGTGAG
7	950A > T-F	GCAGGGAGCGTGGtCACGACGGTGTTTC
	950A > T-R	GAAACACCGTCGTGaCCACGCTCCCTGC
8	1150C > G-F	CTGTTTCTGGAGgGAGTGGCGAGC
	1150C > G-R	GCTCGCCACTCcCTCCAGAAACAG
9	1358G > A-F	GCCAGTGCCTTGGGCaGCGCCTGGCAGAGG
	1358G > A-R	CCTCTGCCAGGCGCtGCCCAAGGCACTGGC
10	1360C > T-F	GTGCCTTGGGCGGtGCCTGGCAGAGGC
	1360C > T-R	GCCTCTGCCAGGCaCCGCCCAAGGCAC

### Site-directed mutagenesis

A pcDNA3.1 expression vector construct with *CYP11B1* complementary DNA (cDNA) as the insert (pcDNA3.1-CYP11B1 construct) was used as previously described. Single point mutations were introduced into *CYP11B1* cDNA in the pcDNA3.1-*CYP11B1*-FLAG construct using PCR-based, site-directed mutagenesis and confirmed by first-generation sequencing.

### Enzymatic activity assay

We studied the enzymatic activity of the 10 *CYP11B1* mutants identified in this study, including some mutations that have been previously analyzed for function or have been reported but not functionally analyzed (Table 2). We resuscitated COS-7 cells were transfected using Lipofectamine 2000 reagent (Invitrogen, USA) with 1 µg of empty plasmid, wild-type *CYP11B1* plasmid, or mutant *CYP11B1* plasmid. After 48 hours of transfection, 1 mL of 11-deoxycortisol at 0.25, 0.5, or 1.0 μmol was added and incubated for 24 hours. The culture supernatant was collected, and the cortisol concentration was detected with an enzyme linked immunosorbent assay (ELISA) kit.

**Table 2:** Clinical characteristics and genotypic of 10 Chinese patients with 11β-OHD

Patients	Karyotype	Age (y)	CA (y)	BA (y)	Ht (SDS)	Wt (SDS)	BP (mmHg)	Prader	External genitalia	Clinical characteristics	Homozygous/heterozygous	cDNA	Protein	Exon
P1	46,XY	4	4.25	13	4	3	98/65	V	Penis 4 × 2 cm, Testis 3 mL, PH2	Hyperpigmentation, premature adrenal, PPP	Compound heterozygous	c.945C > A c.363_365del	p. S315R, p. Q121del	Exon5, exon2
P2	46,XY	8	13	17	1.1	2	150/90	V	Penis 5 × 2 cm Testis 3.5 mL PH5	Hyperpigmentation, premature adrenal, PPP	Compound heterozygous	c.1360C > T Exon3-4del	p. R454C, exon3-4del	Exon8, exon3–4
P3	46,XY	3	8	10	1.7	1.9	140/100	V	Penis 6 × 3 cm Testis 12 mL PH5	Hyperpigmentation, premature adrenal, PPP Hypokalemic and hypertension	Homozygous	c.950A > G	p. D317V	Exon5
P4	46,XY	11	11.58	15	2.27	1	180/120	V	Penis 8 × 3 cm Testis 10 mL PH5	Hyperpigmentation, premature adrenal, PPP, hypokalemic and hypertension	Compound heterozygous	c.396-1G > A c.1377-1378del	c.396-1G > A, p. E459fs	Intron2, exon8
P5	46,XY	11	12	16	1.79	2	150/90	V	Penis 6 × 2 cm Testis 13 mL PH5	Hyperpigmentation, Premature adrenal, PPP, hypokalemic and hypertension	Compound heterozygous	c.1355A > G/ c.1360C > T	p. R453Q, p. R454C	Exon8
P6	46,XX	0	7.2	-	0.16	0.5	91/49	II	B1PH1	Hyperpigmentation, severe virilization	Compound heterozygous	c.1391_1393delTGC c.858C > A	p. L464del, p. Y286X	Exon8, exon5
P7	46,XX	0	4.01	4	− 0.31	− 0.1	96/66	II	B1PH1	Hyperpigmentation, severe virilization	Compound heterozygous	Exon3-4del c.1150-1153delCGAG	Exon3-4del, p. R384Wfs*45	Exon3-4del, exon7
P8	46,XX	0	0.75	–	1.6	1.3	84/51	II	B1PH1	Hyperpigmentation, severe virilization	Homozygous	c.456C > G	p. N152K	Exon3
P9	46,XX	0	1.33	-	1.88	3.1	85/55	II	B1PH1	Hyperpigmentation, severe virilization	Compound heterozygous	c.421C > T c.1360C > T	p. R141X, p. R454C	Exon3, exon8
P10	46,XX	0	3.75	10	4.22	3	125/75	II	B1PH1	Hyperpigmentation, severe virilization	Homozygous	c.1360C > T	p. R454C	Exon8

*11β-OHD* 11beta-hydroxylase deficiency, *CA* actual age, *BA* bone age, *Ht* height, *Wt* weight, *BP* blood pressure, *PH* pubic hair, *PPP* peripheral precocious puberty, *cDNA* complementary DNA

### Western blots

COS-7 cells transfected with empty, wild-type *CYP11B1*, or mutant *CYP11B1* plasmid were lysed in radio immunoprecipitation assay (RIPA) buffer (50 mmol/L Tris–Cl, 150 mmol/L NaCl, 5 mmol/L EDTA, 0.1% sodium dodecyl sulfate (SDS), and 1% NP-40 pH 7.6) supplemented with a protease inhibitor cocktail (Roche, Switzerland). The lysates were denatured at 100 °C for 10 minutes in 1 × SDS- polyacrylamide gel electrophoresis (PAGE) loading buffer, subjected to SDS-PAGE and transferred to a polyvinylidene fluoride (PVDF) membrane (Bio-Rad, USA). The membrane was incubated with appropriate antibodies against FLAG (1:2000, 20,543–1-AP, Proteintech, USA) or glyceraldehyde-3-phosphate dehydrogenase (GAPDH) (1:5000, 60,004–1-Ig, Proteintech, Chicago, USA). The secondary antibodies used were labeled with horse radish peroxidase (HRP), and the signals were visualized using a Tanon 5200 Imaging System (Tanon, Shanghai, China).

### Construction of a three-dimensional structure prediction model of CYP11B1 protein

*CYP11B1* missense mutations can affect the three-dimensional structure of the CYP11B1 enzyme, especially the three-dimensional conformation of the active site, resulting in decreased enzyme activity. Simulation of the three-dimensional structure of the protein at the above mutation sites may help to rationally explain and further confirm the results of the in vitro protein expression experiments.

The amino acid sequences of wild-type and mutant CYP11B1 were introduced into I-TASSER software to simulate the three-dimensional structure of the *CYP11B1* variant protein with the X-ray structure of human CYP11B1 as a template [protein database (PDB accession number: 6M7X)] with visualization using PyMOL software.

### Statistical methods

SPSS 22 was used for statistical analysis. Measurement data are expressed as the mean ± SD (standard deviation) or median. Height deviation was calculated using the Height Standard Deviation Score (SDS), which is calculated as (child height − normal child height)/normal child height SD. The same method was used for calculating weight.

## Results

### Clinical characteristics

The clinical characteristics of the patients are summarized in Table 2. The ten patients (five 46,XY and five 46,XX) were from 10 families. Age at diagnosis was delayed in the 46,XY patients (an average age of 9.77 ± 3.62 years) in comparison to 46,XX patients (an average age of 2.08 ± 1.66 years). All patients had hyperpigmentation. Among the five 46, XY male patients, premature adrenarche and peripheral precocious puberty were present at diagnosis in most cases, and macropenis was present in all male patients. Cases 3–5 involved hypokalemia and hypertension. As all female patients showed masculinization of external genitalia at birth, the age of treatment was earlier. Corticoid treatment consisted of hydrocortisone at diagnosis and during follow-up.

### Hormone level characterization

The Hormonal level of the patients are summarized in Table 3. Seven patients were referred for androgen excess, and three were referred for hypertension and hypokalemia. Before treatment, adrenocorticotropic hormone (ACTH), cortisol, testosterone, aldosterone, renin, and electrolytes (including sodium and potassium) were detected in all patients, and dehydroepiandrosterone sulfate (DHEAS) and androstenedione (AD) were detected in six patients, and 17-OHP was detected in all but one patient. ACTH, 17-OHP, and the aldosterone/renin ratio were all elevated in all 10 patients without treatment; 5 patients had hypokalemia, and 2 patients had hypernatremia. An ACTH stimulation test was performed for 1 child (P9) without medication, the results showed that the baseline value of cortisol was low, The peak value of provocation was still low. The baseline value and peak value of 17-OHP and DHEAS were significantly higher than the normal range, and the baseline value and peak value of AD were also significantly increased.

**Table 3:** Laboratory characteristics in 10 Chinese patients with 11β-OHD

Patients	ACTH (pg/ml)	F (ug/dl)	17-OHP (ng/ml)	Ald (ng/dl)	Renin (ng/ml/h)	Aldrenin	K (mmol/l)	Na (mmol/l)	DHEAS (ug/dl)	AD (ng/ml)	T ng/ml
Reference	7–65	6.7–22.6	0.07–1.53	29.4–161.5	0.1–5.5	< 30	3.5–5.1	130–147	5–57	0.15–3.1	< 0.08
P1	280.5	4.89	109	3.91	0.009	433	3.04	150	–	–	1.55
P2	> 2000	2.27	3.73	28.75	0.27	106	3.81	142	10	1.45	1.8
P3	> 2000	0.03	1.7	50.09	0.07	715	3.71	–	20.27	15.39	2.34
P4	564	3.82	-	103	1.5	68.6	2.52	153	24.8	-	10.31
P5	793	15.8	1.49	209	0.6	349	3.02	148	2.21	19.39	4.2
P6	107.8	0.61	0.37	131	3.98	32.9	4.63	139	18.7	0.1	< 0.08
P7	> 2000	0.51	25.3	87.3	0.04	2182	3.87	142	–	–	< 0.08
P8	> 2000	19	15	78	0.1	780	3.67	146	15	–	< 0.08
P9	> 2000	15.1	22.2	123.3	0.01	12,330	3.15	144	182.6	12.78	0.54
P10	371.6	2.5	16.27	68.96	0.1	689.6	3.35	149	–	> 10	1.83

*11β-OHD* 11beta-hydroxylase deficiency, *17-OHP* 17-hydroxyprogesterone, *ACTH*adrenocorticotropic hormone, *F* cortisol, *T* Testosterone, *Ald* aldosterone, *K* Potassium, *Na* Sodium, *DHEAS* dehydroepiandrosterone, *AD* androstenedione

### Molecular analysis of the CYP11B1 gene

**Fig. 1:**
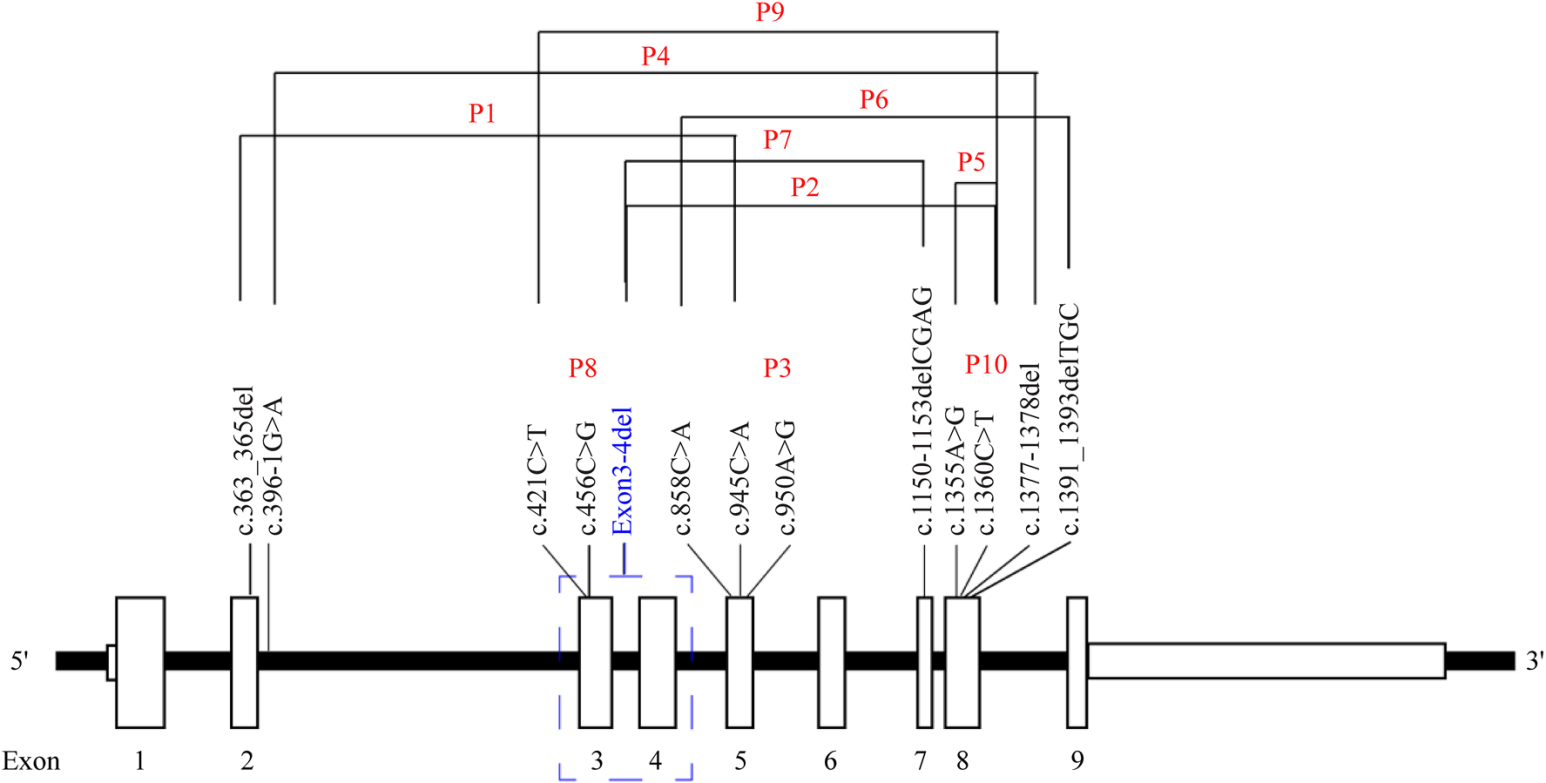
Diagram of the *CYP11B1* gene showing the location of mutations in 10 Chinese patients

Through direct sequencing of PCR products, *CYP11B1* gene mutations were detected in all 10 children; 3 cases involved homozygous mutations, and 7 were compound heterozygous mutations. There were four types of mutations in the 10 children, including four missense mutations (c.945C > A, c.950A > G, c.1355A > G, c.1360C > T), two nonsense mutations (c.421C > T, c.858C > A), five deletion mutations (c.363_365del, c.1391-1393delTGC, exon3-4 del, c.1150-1153delCGAG, c.1377-1378del), and one splice mutation (c.396-1G > A), for a total of 12 unique mutations. Excluding two point mutations and one nonsense mutation that have been described in other reports, these mutations have not been reported in the literature. The mutations included c.363_365del, c.858C > A, c.945C > A, c.950A > G, c.1391-1393delTGC, exon 3–4 del, c.1150-1153delCGAG, c.1377-1378del, and c.396-1G > A mutations. The mutation sites are mainly concentrated in exons 2, 5, 7, and 8. Consistent with previous reports, four patients (2, 5, 9, and 10) carried the c.1360C > T (p. R454C) mutation, which is found only in the Chinese population. The gene mutation results are shown in Table 2. A diagram of the *CYP11B1* gene showing the location of mutations in 10 Chinese patients is provided in Fig. 1.

### Amino acid conservation analysis of new missense mutations

**Fig. 2:**
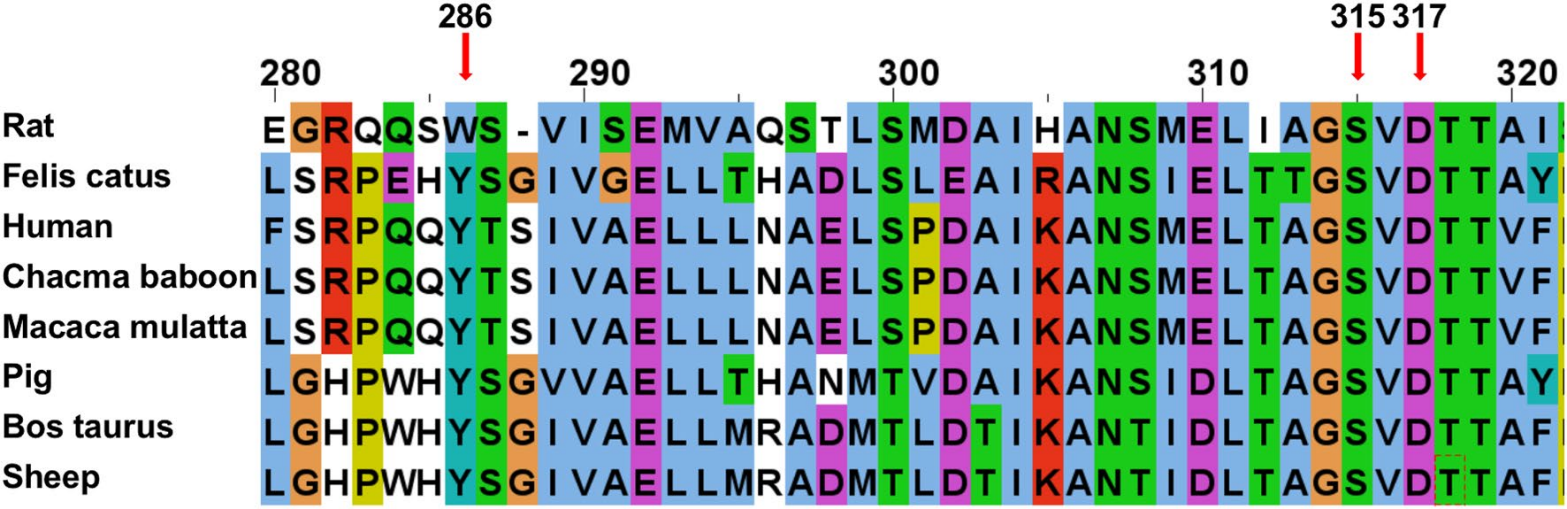
Comparison of the conservation of the missense mutation sites discovered this time among different species *CYP11B1* cytochrome P450 family 11 subfamily B member 1

Through multiple sequence alignment, we found that three new missense mutant amino acid sites of *CYP11B1* to be highly conserved in mammals (Fig. 2), suggesting that mutations at these sites may have an important impact on protein function.

### Identification of mutation sites for functional experiments and protein expression analysis

**Fig. 3:**
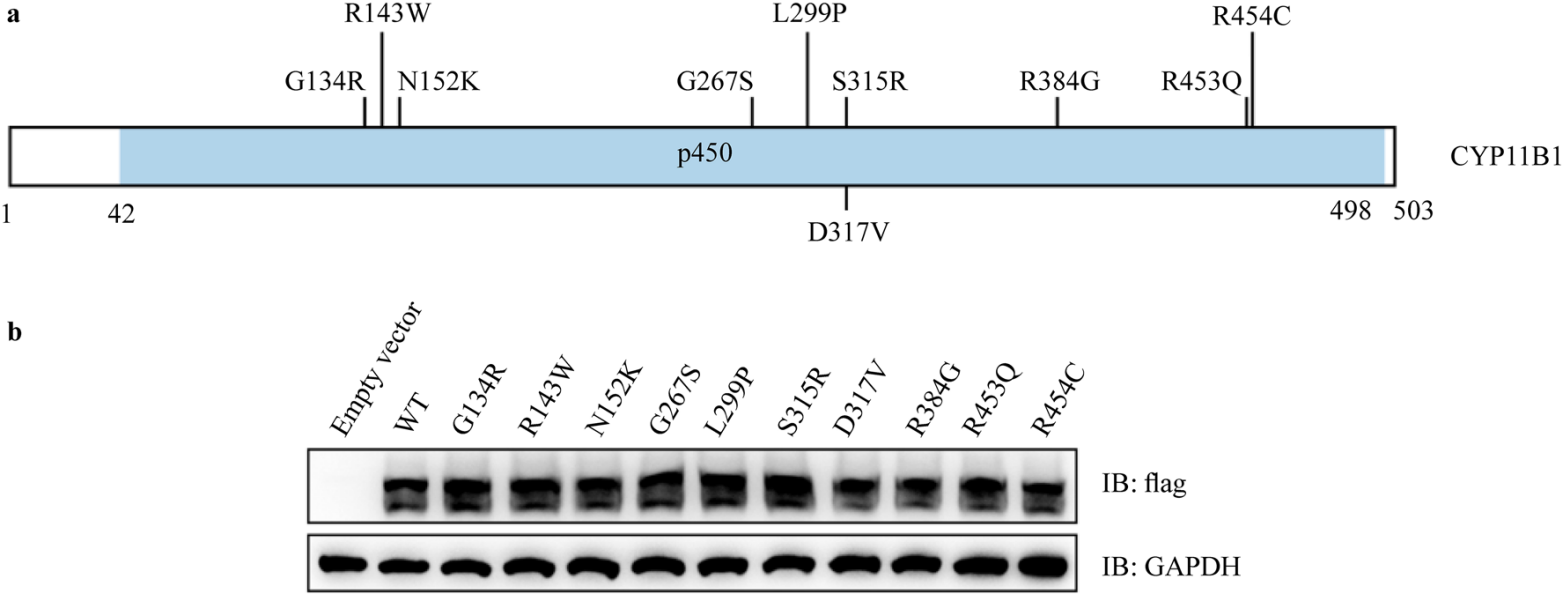
Schematic localization and protein imprinting results of *CYP11B1* mutation. **a** Diagram of these *CYP11B1* genes showing the location of mutations; **b** Western blot results of *CYP11B1* wild-type and mutant. WT wild-type, IB immunoblot, *GAPDH* glyceraldehyde-3-phosphate dehydrogenase

We reviewed relevant literature and summarized new mutations or *CYP11B1* gene mutations that have been reported in the literature but have not been functionally studied, including c.400G > C (p. G134R), c.427C > T (p. R143W), c.456C > G (p. N152K), c.799G > T (p. G267S), c.896 T > C (p. L299P), c.945C > A (p. S315R), c.950A > T (p. D317V), c.1150C > G (p. R384G), c.1358G > A (p. R453Q), and c.1360C > T (p.R454C). Fig. 3a shows the locations of these CYP11B1 gene mutations. Expression levels of wild-type and mutant *CYP11B1* were assessed by western blotting, and no obvious differences were observed (Fig. 3b).

### Residual enzyme activity results

**Fig. 4:**
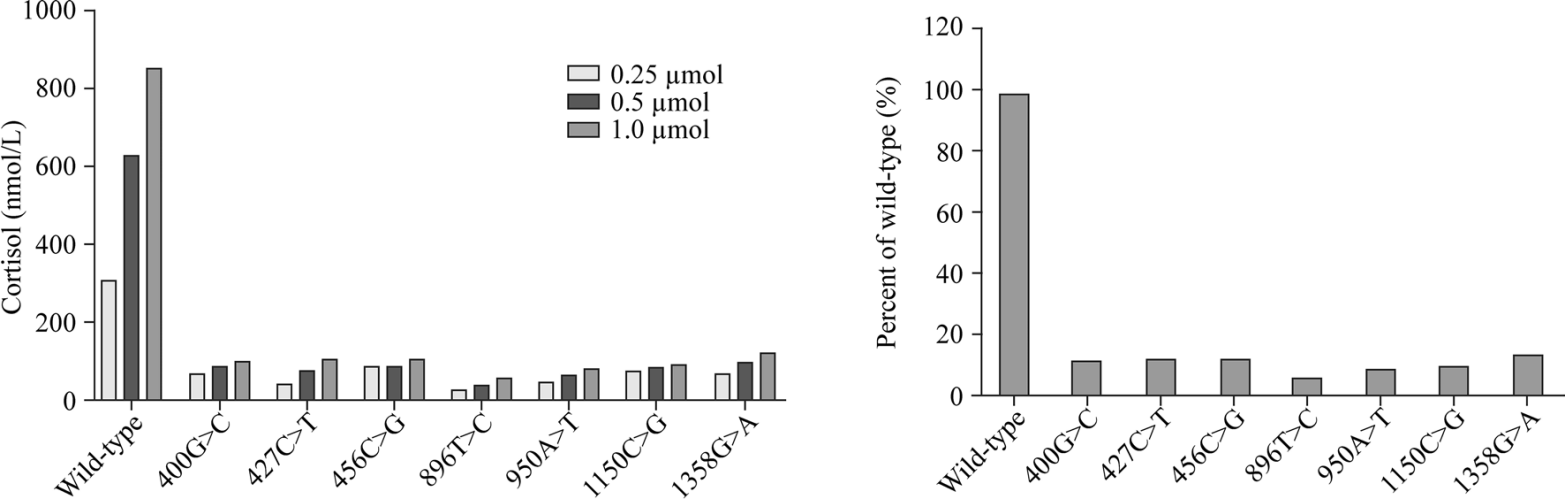
Enzymatic activity assay of wild-type and mutant *CYP11B1*. **a** Production of cortisol in the presence of various concentrations of 11-deoxycortisol (0.25, 0.5, 1.0 µmol) is shown as a measure of 11-hydroxylase activity in COS-7 cells transfected with the plasmid expressing the wild-type protein. **b** Compared with the wild-type enzyme, the 11-deoxycortisol (1 µmol/L) conversion percentages of the mutant enzymes were 11.5%, 12.3%, 12.3%, 6.7%, 9.4%, 10.4%, and 13.9% for c.400G > C (p. G134R), c.427C > T (p. R143W), c.456C > G (p. N152K), c.896 T > C (p. L299P), c.950A > T (p. D317V), c.1150C > G (p. R384G), c.1358G > A (p. R453Q), respectively

The results of the enzymatic activity assay for the wild-type and CYP11B1 mutants proteins are shown in Fig. 4. The wild-type enzymatic activity was defined as 100%. The conversion percentages of 11-deoxycortisol (1 µmol/L) conversion were 11.5%, 12.3%, 12.3%, 6.7%, 9.4%, 10.4%, and 13.9%, respectively.

### *CYP11B1* mutation function prediction and three-dimensional protein structure prediction model

The amino acid conservation analysis of *CYP11B1* in 7 species was carried out through the Uniprot database, and it was found that S315 does not exhibit high conservation; N152 is a variable amino acid; D317 and R454 are highly conserved residues. Then we used PolyPhen-2, SIFT and CADD software to predict whether these four different missense mutation would be harmful to the protein function. The results showed that S315R, D317V and R454C are damaging, while N152K is benign (Table 4).

**Table 4:** Prediction of mutation function of CYP11B1

Patients	Amino acid	Mutation	Activity	PolyPhen-2	SIFT	CADD	Destructive
P1	S315R	Novel missense mutation	Not highly conserved	1.000	0.073	17.80	Damaging
P8	N152K	Missense mutation	Variable	0.000	1.000	0.024	Benign
P3	D317V	Missense mutation	Highly conserved	0.999	0.000	24.3	Damaging
P3, P7, P9, P10	R454C	Missense mutation	Highly conserved	1.000	0.000	25.6	Damaging

*CYP11B1* cytochrome P450 family 11 subfamily B member 1, *PolyPhen-2* Polymorphism Phenotyping v2, *SIFT* sorts intolerant from tolerant, *CADD* Computer-Aided Drug Design

**Fig. 5:**
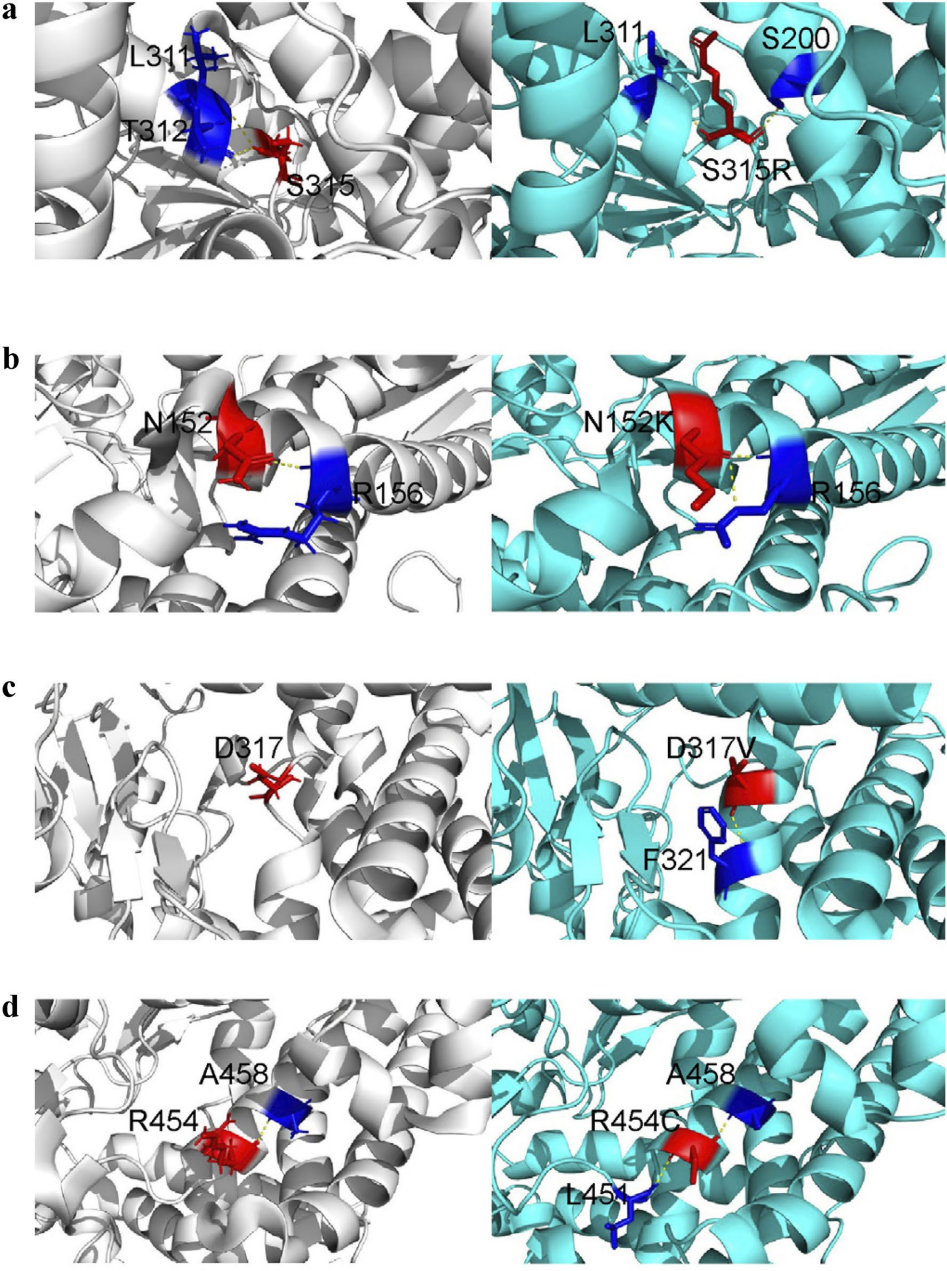
*CYP11B1* three-dimensional protein structure prediction model. White: wild-type; cyan: mutant; red: mutation site. Left: wild type, right: mutant

Figure 5 is a three-dimensional protein structure prediction model for *CYP11B1*. The protein model prediction showed that the p.S315R mutant did not form hydrogen bonds with T312 but increased the hydrogen bonds with S200 (Fig. 5a); the p.N152K mutant was the same as the wild type, and both only formed hydrogen bonds with R156 (Fig. 5b); the p.D317V increases hydrogen bonding with F321, which may enhance the local interaction strength and reduce the flexibility of the domain (Fig. 5c); and p.R454C increases hydrogen bonding with L451, which may enhance local interaction strength and reduce domain flexibility (Fig. 5d).

## Discussion

In this study, we describe the clinical and molecular characterization of 11β-hydroxylase deficiency and the genotype–phenotype correlation of 11β-OHD in 10 Chinese patients from 10 unrelated families. Five female patients were affected by excessive fetal adrenal androgens in the uterus, resulting in ambiguous external genitalia and varying degrees of masculinization at birth [5]. Therefore, the female patients were treated at an early age, at an average age of 2.08 ± 1.66 years old. The age of the 46,XY patients at the time of diagnosis ranged from 4.25 to 12 years, with the difference between bone age and chronological age being 3.69 ± 2.82 years. The age of diagnosis and treatment of female patients was significantly earlier than that of male patients. All 10 patients displayed different degrees of skin pigmentation. Four patients were initially misdiagnosed with 21-OHD. Due to the increase in DOC, some patients develop hypertension with low aldosterone and low renin with hypokalemia. Therefore, the levels of DOC and deoxycortisol are important diagnostic criteria for 11β-OHD. Unfortunately, they are not routinely detected in  China. In our clinical practice, hypertension is the main clue to differentiate 11β-OHD from 21-OHD. However, most patients do not have manifestations of hypertension and hypokalemia at birth, and some patients do not have hypertension [6, 7]. Therefore, it is generally detected later in childhood or even in adolescence. In our study, three male patients presented hypertension and hypokalemia due to delayed diagnosis of 11β-OHD and delayed treatment. The female patients were diagnosed at an early age and were treated in a timely manner without hypertension or hypokalemia. Complications such as cardiomyopathy, retinal vein occlusion, and even blindness from long-lasting, uncontrolled hypertension have been reported in patients with 11β-OHD [8]. Early diagnosis and treatment are key points to prevent the complications of hypertension. The lack of negative feedback of glucocorticoids leads to increased secretion of ACTH, such that adrenal crisis rarely occurs, but patients generally have hyperpigmentation. Therefore, molecular analysis of the *CYP11B1* gene is particularly important to confirm a suspected clinical diagnosis of 11β-OHD.

11β-OHD is caused by mutations in *CYP11B1* located on chromosome 8q21-22. The *CYP11B1* gene consists of nine exons and encodes a 503 amino acid protein. To date, the HGMD has reported 98 missense and nonsense mutations. These mutations are concentrated in exons 2 and 6–8, with a small number in exons 1 and 9. In this study, we detected 13 mutations, including three point mutations and one nonsense mutation that have been described in other reports [9], of which nine mutations are novel (Y286X, S315R, D317V, Q121del, L464del, exon 3–4 deletion, R384Wfs × 45, E459fs, c.396-1G > A). The mutation sites are mainly concentrated in exons 2, 5, 7, and 8. Consistent with previous reports, four patients (P2, P5, P9, P10) carried the R454C pathogenic variation, which is found only in the Chinese population [10]. The nine novel mutations included two missense mutations (S315R and D317V), one nonsense mutation (Y286X), five deletion frameshifts (Q121del, L464del, exon 3–4 deletions, R384Wfs × 45 and E459fs) and one splice mutation (c.396-1G > A). Nonsense mutations, deletion frameshift mutations and splicing mutations all indicate loss of enzymatic activity. Therefore, newly discovered and previously reported missense mutations that have not been tested for enzymatic activity were selected for enzymatic activity function detection, and the encoded enzymatic protein after gene mutation was analyzed. For the D317V mutation detected in patient 4, enzyme activity was 9.4% (< 10%); the patient showed clinically accelerated growth with peripheral precocious puberty, which was misdiagnosed as 21-OHD in other hospitals, and developed hypertension and hypokalemia several years later. Therefore, the disease was considered to be classic 11β-OHD. For patient 8 (46, XX), N152K mutant enzyme activity was 12.3%, this patient had obvious masculinization of external genitalia at birth with renin in infancy of 0.1 ng/mL/h, and serum potassium of 3.67 mmol/L. Therefore, the case was also considered classical 11β-OHD. In 2010, Parajes et al. [11] detected M88I and P159L mutations in patients with the nonclassical form; these mutations decreased the enzyme activity to 40% and 25% of the wild-type level, respectively. While studies have shown that *CYP11B1* mutations that cause classic 11β-OHD usually reduce enzyme activity to less than 5% or result in complete loss of activity, for each specific mutation, there is no significant correlation with the severity of the clinical manifestations for each specific mutation. Indeed, patients with the same gene pathogenic variant may have mild or severe hypertension, and the manifestations of androgen excess vary in severity. Thus, the correlation between genotype and phenotype needs to be further studied.

Through the three-dimensional protein structure model, we attempted to infer the influence of the mutation site on the three-dimensional structure of the protein conformation. The helix structure is the heme-binding region and is highly conserved. The cysteine sulfhydryl group at position 450 binds with a heme iron atom to form one of the enzyme active sites. Therefore, the amino acid changes around C450 can affect binding of heme and cause the loss of enzyme activity. For example, Y423X, Q426X, P427H, V441G, G444D, G446V, G446S, R448C, R448H, R448P, R453Q, R454C, R461P and other mutations can cause a loss of enzyme activity [10, 12], of which R454C has been reported only in the Chinese population. The R454 residue is highly conserved; protein model predictions show increased hydrogen bonding with L451 for the p.R454C mutant, which may enhance local interaction strength and reduce domain flexibility.

As part of the substrate binding pocket, the I-helix contains many hydrophobic amino acids and potential enzyme active sites and is involved in the recognition of and binding to substrates. When its conformation changes, the enzyme activity of CYP11B1 may be impaired. For example, L299P, A368D, P94L, A331V, E371G and other mutations can reduce or even eliminate the activity of the enzyme [13–15]. The G-helix, the I-helix and the B-C loop between them are important channels for the substrate to enter the active site the G-helix is necessary for the initial recognition of the substrate, and the change in the hydrophobic environment around it can hinder the binding of the substrate to the enzyme. For example, W116C, W116G, V129M, V148G, A259D, T318M, T318R, T318P, R384Q, R384G and other mutations can reduce or even cause loss of enzyme activity [15]. The c.945C > A mutation was detected in patient 1, resulting in the amino acid change S315R (Ser315Arg), which is a new missense mutation. The amino acid conservation analysis of CYP11B1 in seven species was carried out through the UniProt database, and S315 is not highly conserved; PolyPhen-2 (score 1.000) software predicts the p. S315R variant to be deleterious, but with SIFT (score 0.073) and CADD (score 17.80) software, the deleterious threshold was not reached. The protein model prediction showed that the p.S315R mutant does not form hydrogen bonds with T312 but shows increased the hydrogen bonding with S200. The c.456C > G mutation was detected, leading to the amino acid N152K (Asn315Lys), was detected in patient 8. This missense mutation has been reported, but no functional experiments have been performed.Amino acid conservation analysis of CYP11B1 in seven species was performed through the UniProt database. N152 is a variable amino acid; PolyPhen-2 (score 0.000), SIFT (score 1.000), and CADD (score 0.024) predict that the p. N152K variant to be benign. The protein model prediction showed that the p. N152K mutant folds in the same manner as the wild-type protein, and in both cases, the residue at this position forms hydrogen bonds only with R156. This patient is 46,XX, with clinical manifestations of clitoral hypertrophy, visible vaginal opening, urethral opening, Prader stage II, and Tanner stage B1PH1, but no manifestations of hypertension. Therefore, this current missense mutation of CYP11B1 can lead to changes in the three-dimensional conformation of the CYP11B1 enzyme, leading to different degrees of changes in enzyme activity.

In conclusion, c.1360C > T is the most common *CYP11B1* pathogenic variant in the Chinese population. Enzymatic activity assays combined with clinical characteristics showed a good clinical phenotype-genotype correlation in this study. Three-dimensional protein simulations may provide additional support for the physiopathological mechanism of genetic mutations. Further research is still needed in the future.

## Data Availability

All data generated or analyzed during this study are included in this published article.
